# Nuclear S100A7 Is Associated with Poor Prognosis in Head and Neck Cancer

**DOI:** 10.1371/journal.pone.0011939

**Published:** 2010-08-03

**Authors:** Satyendra Chandra Tripathi, Ajay Matta, Jatinder Kaur, Jorg Grigull, Shyam Singh Chauhan, Alok Thakar, Nootan Kumar Shukla, Ritu Duggal, Siddhartha DattaGupta, Ranju Ralhan, K. W. Michael Siu

**Affiliations:** 1 Department of Biochemistry, All India Institute of Medical Sciences, New Delhi, India; 2 Department of Chemistry, York University, Toronto, Ontario, Canada; 3 Centre for Research in Mass Spectrometry, York University, Toronto, Ontario, Canada; 4 Department of Mathematics and Statistics, York University, Toronto, Ontario, Canada; 5 Department of Otorhinolaryngology, All India Institute of Medical Sciences, New Delhi, India; 6 Department of Surgery, Dr. B. R. A. Institute Rotary Cancer Hospital, All India Institute of Medical Sciences, New Delhi, India; 7 Department of Dental Surgery, All India Institute of Medical Sciences, New Delhi, India; 8 Department of Pathology, All India Institute of Medical Sciences, New Delhi, India; 9 Joseph and Mildred Sonshine Family Centre for Head and Neck Diseases, Mount Sinai Hospital, Toronto, Ontario, Canada; 10 Department of Otolaryngology – Head and Neck Surgery, Mount Sinai Hospital, Toronto, Ontario, Canada; 11 Department of Pathology and Laboratory Medicine, Mount Sinai Hospital, Toronto, Ontario, Canada; 12 Department of Otolaryngology – Head and Neck Surgery, University of Toronto, Toronto, Ontario, Canada; Karolinska Institutet, Sweden

## Abstract

**Background:**

Tissue proteomic analysis of head and neck squamous cell carcinoma (HNSCC) and normal oral mucosa using iTRAQ (isobaric tag for relative and absolute quantitation) labeling and liquid chromatography-mass spectrometry, led to the identification of a panel of biomarkers including S100A7. In the multi-step process of head and neck tumorigenesis, the presence of dysplastic areas in the epithelium is proposed to be associated with a likely progression to cancer; however there are no established biomarkers to predict their potential of malignant transformation. This study aimed to determine the clinical significance of S100A7 overexpression in HNSCC.

**Methodology:**

Immunohistochemical analysis of S100A7 expression in HNSCC (100 cases), oral lesions (166 cases) and 100 histologically normal tissues was carried out and correlated with clinicopathological parameters and disease prognosis over 7 years for HNSCC patients. Overexpression of S100A7 protein was significant in oral lesions (squamous cell hyperplasia/dysplasia) and sustained in HNSCC in comparison with oral normal mucosa (p_trend_<0.001). Significant increase in nuclear S100A7 was observed in HNSCC as compared to dysplastic lesions (p = 0.005) and associated with well differentiated squamous cell carcinoma (p = 0.031). Notably, nuclear accumulation of S100A7 also emerged as an independent predictor of reduced disease free survival (p = 0.006, Hazard ratio (HR = 7.6), 95% CI = 1.3−5.1) in multivariate analysis underscoring its relevance as a poor prognosticator of HNSCC patients.

**Conclusions:**

Our study demonstrated nuclear accumulation of S100A7 may serve as predictor of poor prognosis in HNSCC patients. Further, increased nuclear accumulation of S100A7 in HNSCC as compared to dysplastic lesions warrants a large-scale longitudinal study of patients with dysplasia to evaluate its potential as a determinant of increased risk of transformation of oral premalignant lesions.

## Introduction

Head and neck squamous cell carcinoma (HNSCC) is the sixth most common cancer accounting for over 500,000 new cases annually worldwide that includes sites in the oral cavity, pharynx and larynx [1]. Squamous cell carcinoma of the oral cavity accounts for two-thirds of the HNSCC cases occurring in developing countries. The majority of oral squamous cell carcinomas are preceded by visible changes of the oral mucosa. Leukoplakia is the most commonly encountered oral lesion of the oral cavity. These oral leukoplakia lesions show histological evidence of squamous cell hyperplasia or dysplasia. The oral lesions with histologically confirmed dysplasia are termed as oral premalignant lesions (OPLs); on average, about one percent of oral lesions transform into cancer annually [2]–[4]. Despite improvement in treatment strategies, including surgery, radiotherapy (RT) and/or chemotherapy (CT), the prognosis of OSCC patients remains largely unsatisfactory, due to loco-regional recurrence. The 5-year survival rate is less than 50%, and the prognosis of advanced cases has not improved much over the past three decades [5], [6]. At present, the most important prognostic factors include histological tumor grade, stage, depth of the tumor invasion, and involvement of regional lymph nodes at the time of diagnosis. In addition to these clinicopathological parameters, molecular markers are being intensively sought and verified for this malignancy. Lack of biomarkers for early detection and risk assessment is clearly reflected by the fact that more than 50% of all HNSCC patients have advanced disease at the time of diagnosis [5].

In our recent study using iTRAQ (isobaric tag for relative and absolute quantitation) labeling and multidimensional liquid chromatography/tandem mass spectrometry (LC-MS/MS) for examining differential protein expressions between HNSCC and non-malignant tissues, we identified a panel of biomarker candidates for this malignancy [7]. S100A7/psoriasin was identified as overexpressed in HNSCC and emerged among the panel of three best-performing potential biomarkers for distinguishing HNSCC from normal oral mucosa [7]. In another independent study using iTRAQ, we also reported increased expression of S100A7 protein in oral premalignant lesions (dysplasia), albeit in only limited number of cases [8].

S100 protein family consists of at least 25 different types of low molecular-weight proteins (9–13 kDa), which are characterized by the presence of two calcium-binding sites of the EF-hand type conformation [9]–[12]. S100A7 gene is located within the ‘epidermal differentiation complex’ on human chromosome 1q21 [13]–[16]. S100A7 protein , with a molecular weight of 11.4 kDa, was found to be upregulated in skin lesions of psoriatic patients [17]. S100A7 is distributed in the cytoplasm of keratinocytes in normal human epidermis and is present at the cell periphery in terminally differentiated keratinocytes [18]. Increased S100A7 expression has been reported in several epithelial malignancies such as, in situ ductal breast carcinoma, lung, bladder, skin, esophageal and gastric cancer [19]–[24]. Altered expression of S100A4 and S100A2 proteins has been associated with prognosis in HNSCC [10], [25]–[28]. S100A7 overexpression has also been reported in a small set of HNSCC [29], [30]. Although increased expression of S100A7/psoriasin has been reported in these studies, the impact of its expression on cancer development, disease prognosis, and survival of HNSCC patients remains to be completely determined. In this context our study assumes importance, because of its retrospective nature, the large set of patients representing different stages of HNSCC, and the long term follow-up analysis. We analyzed the expression of S100A7/psoriasin in HNSCC, oral lesions (with histological evidence of squamous cell hyperplasia or dysplasia) and non-malignant oral tissues by immunohistochemistry, determined its correlation with clinicopathological parameters, and investigated its utility as a prognostic marker for HNSCC.

## Results

### Immunohistochemical analysis of S100A7 expression in oral leukoplakia lesions and cancers

**Figure 1:**
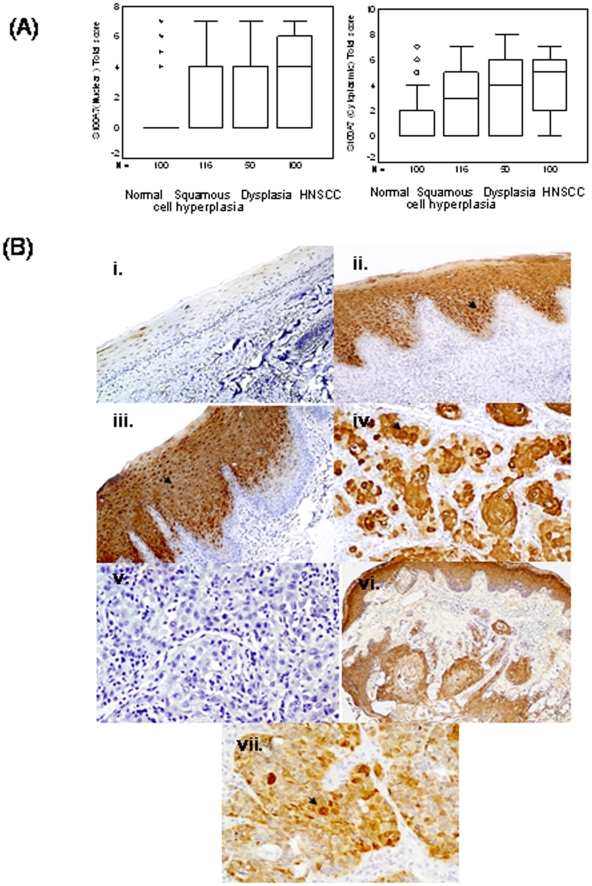
(A) Box-Plot analysis: Box plots showing distribution of total scores based on immunohistochemistry of S100A7 protein in paraffin-embedded sections of oral normal tissues, squamous cell hyperplasia, dysplasia and HNSCC. The vertical axis shows the total immunostaining score, obtained as described in the Methods section. (i) Nuclear S100A7 expression in squamous cell hyperplasia (IHC scoring range 0–7), dysplasia (range 0–7) and HNSCC (range 0–7) (b) cytoplasmic S100A7 in normal (range 0–7), squamous cell hyperplasia (range 0–7), dysplasia (range 0–7) and HNSCC (range 0–7). **(B)** **Immunohistochemical analysis of S100A7 in head-and-neck tissues. Paraffin-embedded sections of histologically normal mucosa, squamous cell hyperplasia or with dysplasia, and HNSCC were stained using anti-S100A7 monoclonal antibody as described in the Methods section.** (i) Normal oral mucosa showing no S100A7 immunostaining; (ii) squamous cell hyperplasia showing nuclear and cytoplasmic S100A7 immunostaining; (iii) dysplasia depicting nuclear and cytoplasmic S100A7 immunostaining in epithelial cells; (iv) HNSCC illustrating both intense cytoplasmic and nuclear staining in tumor cells; (v) HNSCC section with a dysplasia showing S100A7 immunostaining in epithelial cells (original magnification ×100); (vi) HNSCC used as a negative control, showing no S100A7 immunostaining in tumor cells; and (vii) ER-negative breast cancer tissue showing S100A7 immunostaining. Arrows show nuclear and cytoplasmic localization (i-v, vii original magnification ×200).

To determine the clinical significance of S100A7 protein in head-and-neck tumorigenesis, its expression was analyzed in clinical specimens from HNSCC, oral leukoplakia lesions with squamous cell hyperplasia or dysplasia, and histologically normal tissues, using a specific monoclonal antibody by immunohistochemistry. Figure 1A shows the total immunostaining score distribution of nuclear/cytoplasmic S100A7 expression in oral normal tissues, oral leukoplakia lesions with squamous cell hyperplasia or dysplasia and HNSCC. Of the 100 normal tissues analyzed, 84% did not show detectable S100A7 immunostaining in nucleus/cytoplasm of the epithelial cells (Figure 1B(i)). In the remaining normal tissues (16%), moderate cytoplasmic staining was observed in differentiated epithelial cells in the suprabasal layer only. Chi square trend analysis showed significant increase in S100A7 expression (nuclear/cytoplasmic) in tissues obtained from different stages of head-and-neck tumorigenesis (normal, squamous cell hyperplasia, dysplasia and HNSCC; Table 1, p_trend_<0.001).

**Table 1:** Analysis of S100A7 protein expression in Oral Lesions and its correlation with clinicopathological parameters.

Clinicopathological Features	Total Cases	Nuclear N	Positivity (%)	p-value	OR (95% CI)	Cytoplasmic N	Positivity (%)	p-value	OR (95% CI)
**NORMAL**	**100**	**5**	**(5)**			**16**	**(16)**		
**ORAL LESIONS (OL)**	**166**	**62**	**(37.3)**	**<0.001** 5	**11.3 (4.3−29.3)**	**97**	**(58.4)**	**<0.001** 6	**7.4 (3.9−13.7)**
**SQUAMOUS CELL HYPERPLASIA**	116	40	(34.5)	**<0.001** 7	**10.0 (3.8−26.6)**	63	(54.3)	**<0.001** 8	**6.2 (3.2−11.9)**
**DYSPLASIA**	50	22	(44.0)	**<0.001** 9	**14.9 (5.1−43.0)**	34	(68.0)	**<0.001** 10	**11.2 (5.0−24.8)**
**HNSCC**	**100**	**67**	**(67.0)**	**<0.001** 11	**38.6 (14.4−103.9)**	**74**	**(74.0)**	**<0.001** 12	**14.9 (7.4−29.9)**
**Age (Median, 53 yrs) (25–85 yrs)**									
<53	49	31	(63.3)	0.43	---	35	(71.4)	0.566	---
≥53	51	36	(70.6)			39	(76.5)		
**Gender**									
Male	75	51	(68.0)	0.71	---	58	(77.4)	0.188	---
Female	25	16	(64.0)			16	(64.0)		
**Histological Differentiation**									
Well	45	35	(77.8)	**0.031** 13	**2.6 (1.1−6.6)**	37	(84.1)	**0.09**	---
Moderate	49	32	(65.3)			35	(71.4)		
Poor	6	0	(0)			2	(33.3)		
**Tumor Size**									
T_1_+ T_2_	39	23	(59.0)	0.17	---	24	(61.5)	**0.02**	**2.8 (1.1−7.1)**
T_3_ + T_4_	61	44	(72.1)			50	(82.0)		
**Nodal Status**									
N_0_	33	21	(63.6)	0.62	---	22	(66.7)	0.241	---
N_1–4_	67	46	(68.7)			52	(77.6)		
* **Habits**									
Non consumer	22	15	(68.2)	0.89	---	15	(68.2)	0.69	---
Tobacco consumer	78	52	(66.7)			59	(75.6)		

**Nuclear staining**:

5 Normal vs. oral lesions.

7 Normal vs. squamous cell hyperplasia.

9 Normal vs. dysplasia.

11 Normal vs. HNSCC.

13 Well differentiated SCCs vs Moderately and poorly differentiated SCCs; Squamous cell hyperplasia vs dysplasia, p = 0.245; HNSCC vs dysplasia, p = 0.005; N/OL/HNSCC: p_trend_<0.001.

**Cytoplasmic staining**:

6 Normal vs oral lesions.

8 Normal vs squamous cell hyperplasia.

10 Normal vs Dysplasia.

12 Normal vs. HNSCC; N/OL/HNSCC: p_trend_<0.001; Squamous cell hyperplasia vs Dysplasia p = 0.101.

*tobacco consumption habits include tobacco chewing and/or smoking of bidi or cigarettes, chewing of betel quid, areca nut or pan masala.

#### Oral leukoplakia lesions (squamous cell hyperplasia/dysplasia)

Of the 166 oral leukoplakia lesions analyzed, 97 cases (58.4%) showed significant increase in cytoplasmic S100A7 immunostaining (p<0.001, Odds ratio (OR) = 7.4, 95% CI = 3.9−13.7). Among these immunopositive cases 62 tissues showed significant increase in nuclear S100A7 immunostaining also (p<0.001, OR = 11.3, 95% CI = 4.3−29.3) in comparison with the normal tissues (Table 1). These 166 oral leukoplakia lesions included 116 squamous cell hyperplasias; 54.3% (63/116) cases showed significant increase in cytoplasmic S100A7 immunostaining (total score >3, p<0.001, OR = 6.2, 95% CI = 3.2−11.9) relative to the normal tissues (Table 1 and Figure 1B(ii)). Significant increase in nuclear S100A7 immunostaining was also observed in 40/116 (34.5%) cases (p<0.001, OR = 10.0, 95% CI = 3.8−26.6). Notably, increased cytoplasmic localization of S100A7 was observed in 68% dysplasia (34 of 50 cases) (p<0.001, OR = 11.2, 95% CI = 5.0−24.8) in comparison with normal tissues (Table 1 and Figure 1B(iii)). Similarly, progressive increase in nuclear expression of S100A7 was also observed in 22/50 (44%) dysplasia (p<0.001, OR = 14.9, 95% CI = 5.1−43.0). Interestingly, S100A7 overexpression (cytoplasmic/nuclear) was restricted to parabasal and suprabasal layers only. None of these tissue sections showed S100A7 expression in proliferating layers in the basement membrane. Mild membranous S1007 immunostaining (total score <3) was observed in 3 squamous cell hyperplasias, but in none of the dysplasias analyzed.

#### HNSCC

zWe observed a similar pattern of S100A7 expression in HNSCC as well. Sixty seven out of 100 HNSCC (67%) showed nuclear localization of S100A7 in tumor cells as compared to the normal tissues (p<0.001, OR = 38.6, 95% CI = 14.4−103.9). Notably, significant increase in nuclear S100A7 expression was observed in HNSCC (67%) as compared to dysplasia (44%) (p = 0.005, OR = 2.7, 95% CI = 1.3−5.4). In addition to nuclear staining, intense S100A7 staining was also observed in the cytoplasm of tumor cells in 74 of 100 HNSCC analyzed (p<0.001, OR = 14.9, 95% CI = 7.4−29.9, Table 1 and Figure 1B (iv)). The clinicopathological parameters of HNSCC and their correlation with nuclear/cytoplasmic expression of S100A7 are shown in Table 1. Interestingly, nuclear S100A7 overexpression showed an association with histopathological differentiation of HNSCC (p = 0.031). None of the HNSCC tissues showed membranous S100A7 immunostaining. Majority of the HNSCC tissues analyzed in this S100A7 immunohistochemistry study had more than 80% tumor cells in H&E sections. However, there were five cases that showed dysplastic or hyperplastic areas tissue adjacent to the tumor and these regions showed immunostaining similar to that observed in the cases that had only dysplasia or hyperplasia (Figure 1B (v). No immunostaining was observed in HNSCC tissue sections used as negative controls where the primary antibody was replaced by isotype specific IgG (Figure 1B (vi)), while the positive control (ER-negative breast cancer) showed S100A7 expression (Figure 1B (vii)).

### Evaluation of S100A7 as potential diagnostic marker for oral leukoplakia lesions and HNSCC

**Figure 2:**
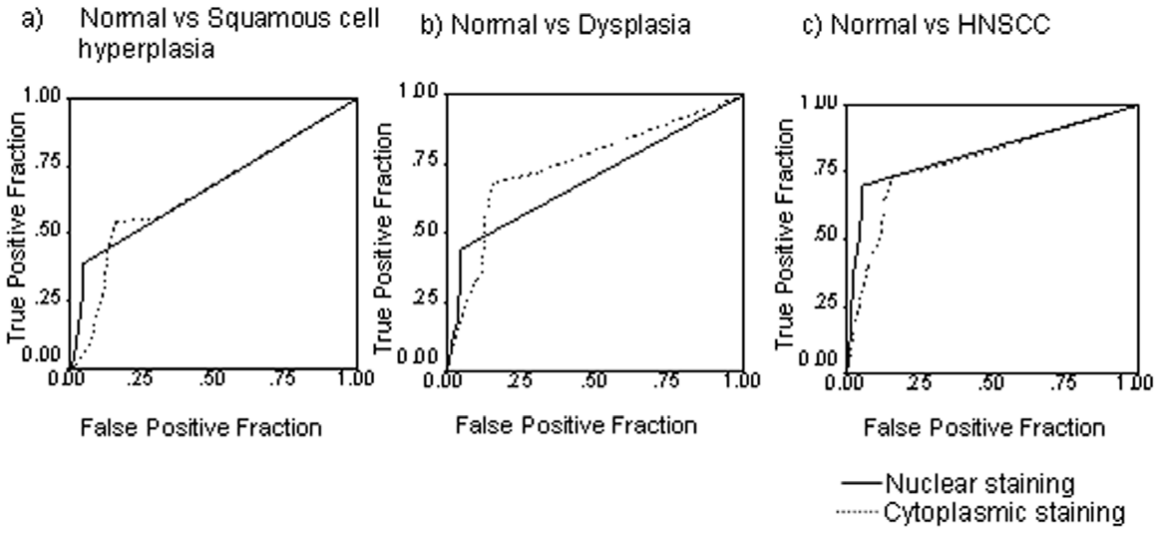
Receiver operating characteristic curves of S100A7 (nuclear/cytoplasmic) in (a) normal vs. squamous cell hyperplasia; (b) normal vs. dysplasia; (c) normal vs. HNSCC. Bold line shows ROC analysis for nuclear S100A7. Dashed line shows ROC analysis for cytoplasmic S100A7. Y-axis of the plot shows true-positive fraction and X-axis shows false positive fraction.

Receiver Operating Characteristic (ROC) analysis was used to determine the potential of S100A7 overexpression to distinguish squamous cell hyperplasia, dysplasia and HNSCC from normal oral tissues. The values for area-under-the-curve (AUC) were 0.664, 0.691 and 0.824 for squamous cell hyperplasia (Figure 2a), dysplasia (Figure 2b), and HNSCC (Figure 2c), respectively (Table 2). Similarly, ROC analysis was used for determination of AUC for cytoplasmic S100A7 staining in all these three groups and area-under-the-curve (AUC) values were 0.650, 0.746 and 0.788 respectively as shown in Table 2 and Figure 2a–2c. The positive predictive values (PPV) were 88.9, 81.5, and 93.1 for nuclear immunostaining. Similarly, for cytoplasmic immunostaining positive predictive values (PPV) were 79.8, 68.0, and 82.2 in the three groups (Table 2).

**Table 2:** Biomarker Analysis of S100A7 (Nuclear/Cytoplasmic) In Oral Lesions.

S100A7	Sensitivity	Specificity	PPV	AUC
**Nuclear Staining**				
Normal vs. squamous cell hyperplasia	34.5	95.0	88.9	0.664
Normal vs. dysplasia	44.0	95.0	81.5	0.691
Normal vs. HNSCC	67.0	95.0	93.1	0.824
**Cytoplasmic Staining**				
Normal vs. squamous cell hyperplasia	54.3	84.0	79.8	0.650
Normal vs. dysplasia	68.0	84.0	68.0	0.746
Normal vs. HNSCC	74.0	84.0	82.2	0.788

### Evaluation of S100A7 overexpression as prognostic marker for HNSCC

**Figure 3:**
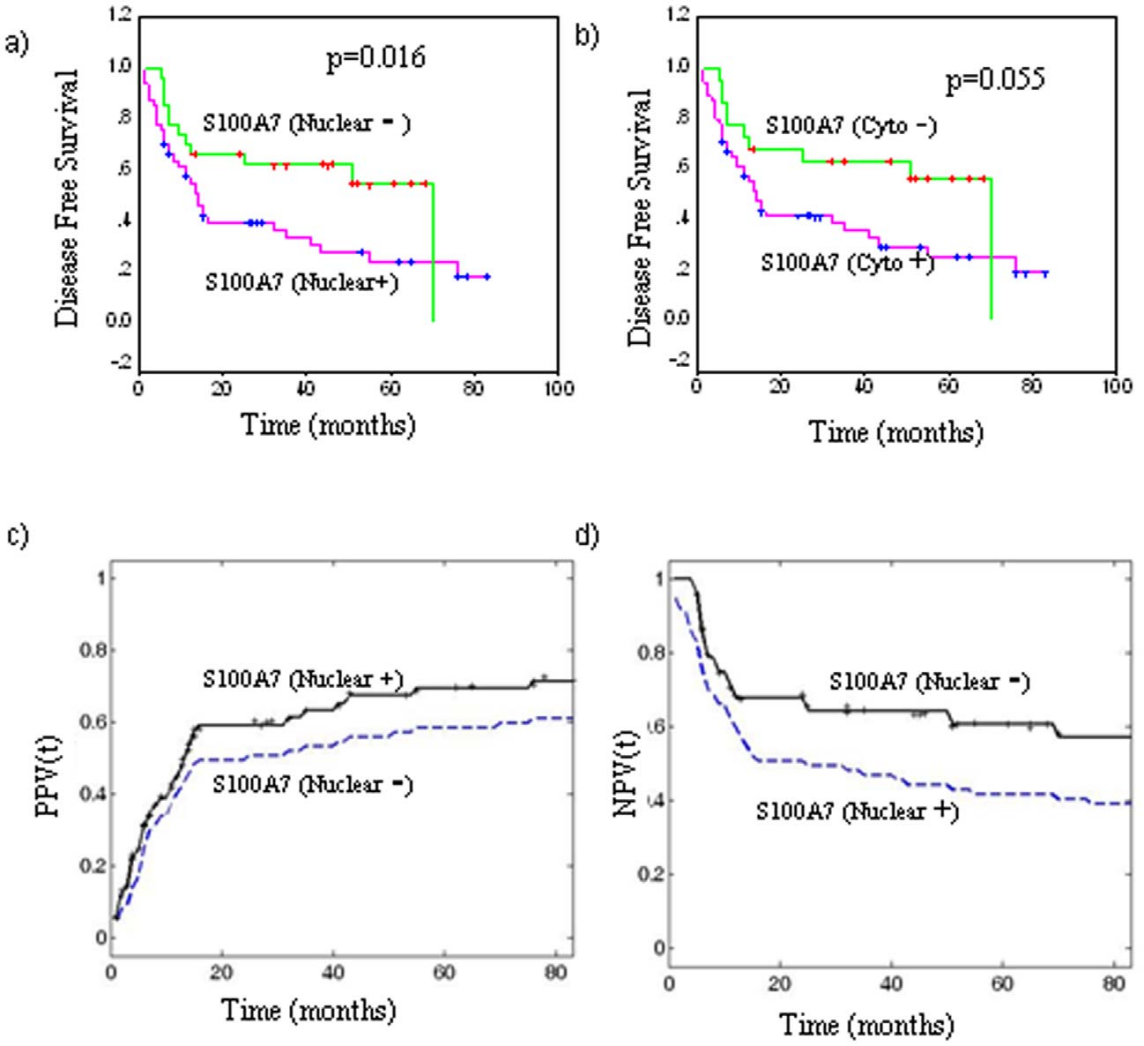
Evaluation of S100A7 overexpression (nuclear/cytoplasmic) as a prognostic marker in HNSCC. Kaplan–Meier estimation of cumulative proportion of disease-free survival: (a) Median time for disease-free survival (DFS; no recurrence/metastasis) in HNSCC patients showing nuclear immunostaining of S100A7 was 13 months, whereas in patients showing no/faint S100A7 immunostaining in nucleus median DFS was 70 months (p = 0.016); (b) In patients showing increased cytoplasmic S100A7 expression the median DFS was 14 months compared with HNSCC that showed mild or moderate cytoplasmic immunostaining (median DFS  = 70 months, p = 0.055). Time-dependent Positive and Negative Predictive Values (PPV(t), NPV(t)) of nuclear S100A7 expression. (c) PPV(t) for time to cancer relapse for 49 HNSCC patients with S100A7^+^ (solid line) and for all 77 HNSCC patients with survival data (dashed line); d) NPV(t) for time to cancer relapse for 28 HNSCC patients with S100A7^−^ (solid line).

The estimated predictive power of the marker i.e. the strength of the statistical association of S100A7 expression with poor prognosis was assessed by Kaplan-Meier survival analysis. Kaplan–Meier survival analysis showed significantly reduced disease-free survival (p = 0.016; median survival 13 months) in HNSCC patients harboring increased nuclear expression of S100A7, compared with median disease-free survival of 70 months in the patients showing no nuclear S100A7 immunostaining (Figure 3a). Similarly, reduced disease-free survival of 14 months was observed in HNSCC patients showing intense cytoplasmic expression of S100A7, compared with patients who did not show increased cytoplasmic S100A7 (median survival of 70 months, Figure 3b). Cox regression analysis was carried out to determine the prognostic potential of S100A7 expression (nuclear/cytoplasmic) for HNSCC in comparison with the other clinical and pathologic parameters - histological grade, tumor size and nodal status (Table 3). Nuclear S100A7 expression emerged as the most significant prognostic marker for HNSCC (p = 0.006, Hazard's ratio (HR)  = 7.6, 95% CI  = 1.3–5.1).

**Table 3:** Correlation of Overall Survival with Clinicopathological Parameters and S100A7 Expression: Multivariate Analysis.

S. No.	Clinicopathological parameter	p-value	HR (95% C.I.)
1	Histological differentiation	**0.003**	**6.6 (1.2−4.1)**
2	S100A7 (nuclear)	**0.006**	**7.6 (1.3−5.1)**

Based on our data, the additional prognostic value that nuclear S100A7 expression provided for predicting cancer recurrence (PPV) in HNSCC patients was measured by the ratio: PPV_relapse/HNSCC_ (83 months|S100A7)/PPV_relapse/HNSCC_ (83 months)  = 71.4/61.0; or for excluding (NPV) cancer recurrence in HNSCC patients was NPV_relapse/HNSCC_ (83 months|S100A7)/NPV_relapse/HNSCC_ (83 months)  = 57.1/39.0, as shown in Figure 3c and 3d respectively.

### Verification of S100A7 overexpression by RT-PCR and Western blotting

**Figure 4:**
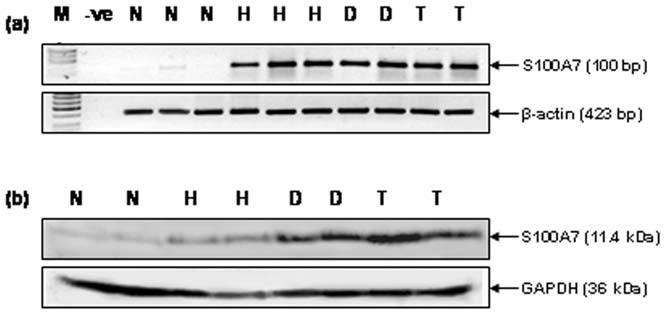
Verification of S100A7 expression in tissues. (a) RT-PCR analysis of S100A7 in oral normal mucosa, squamous cell hyperplasia, dysplasia and HNSCC tissues. For RT-PCR analysis and Western blot analysis, we used normal (n = 5), hyperplasia (n = 5), dysplasia (n = 5) and HNSCC (n = 5) tissues. Panel shows increased levels of S100A7 transcripts in oral lesions -squamous cell hyperplasia (H), dysplasia (D) and HNSCC (T) compared with the oral normal mucosa (N) that showed basal levels of S100A7 transcripts. β-actin used as a control to normalize the quantity of RNA used for each RT-PCR reaction is shown in the lower panel. (b) Western blot analysis of S100A7 in oral normal mucosa (N), squamous cell hyperplasia (H), dysplasia (D) and HNSCC tissues. Equal amount of protein lysates from these tissues were electrophoresed on 12% SDS-PAGE and transferred to PVDF membrane. The membrane was incubated with respective primary and secondary antibodies as described in the Methods section and the signal detected by enhanced chemiluminescence method. Panel shows increased expression of S100A7 protein in oral lesions - squamous cell hyperplasia (H), dysplasia (D) and HNSCC (T) compared with oral normal mucosa (N). GAPDH was used as loading control.

The overexpression of S100A7 in oral lesions was verified by RT-PCR and Western blot analyses in the same representative tissue samples as used for immunohistochemical analysis. RT-PCR analysis demonstrated increased levels of S100A7 transcripts in squamous cell hyperplasia, dysplasia, and HNSCC in comparison with normal tissues (Figure 4a). Western blot analysis showed a single intense band of 11.4 kDa, confirming the increased expression in squamous cell hyperplasia, dysplasia and HNSCC, as compared to the normal tissues (Figure 4b).

## Discussion

The salient findings of our study are: (i) significant increase in S100A7 expression (cytoplasmic/nuclear) in squamous cell hyperplasia, dysplasia and HNSCC in comparison with normal oral tissues; (ii) cytoplasmic S100A7 expression distinguishes squamous cell hyperplasia, dysplasia and HNSCC from normal mucosa with high specificity and PPV; (iii) significant increase in nuclear S100A7 accumulation in HNSCC as compared to dysplasia; and (iv) potential of nuclear S100A7 as a marker of poor prognosis of HNSCC. It is noteworthy that studies on molecular analysis of oral leukoplakia lesions - with squamous cell hyperplasia or dysplasia are very limited, often because these patients do not seek medical attention, due to small lesions that do not pose any serious clinical problems. Increased S100A7 expression has been reported in dysplastic lesions [29], [30]. However, our data suggest S100A7 overexpression as early as in squamous cell hyperplasia with no histological evidence of dysplasia. The onset of squamous cell hyperplasia is often associated with chronic inflammation and the molecular links between inflammation and pre-malignancy are being intensively pursued. In this context, notably earlier studies have reported the role of S100 proteins in inflammation, supporting our findings [21], [31]–[33]. S100A7 was identified in oral pre-malignant epithelia (dysplasia) by microarray analysis and proposed to be a marker for inflammation [34]. S100A7 has been shown to play a role in facilitating the host inflammatory cell response, where it is implicated as a chemotactic factor for lymphocytes and neutrophils in skin disease [35]. S100A7 has also been associated with increased inflammatory cell infiltrates across all types of invasive breast tumors [24]. In addition, S100A7 has been proposed as an epidermal response gene to inflammatory cytokines [36], [37]. In breast cancer (DCIS), both oncostatin M (OSM) and interleukin-6 (IL-6) have been proposed to regulate the expression and activity of S100A7 by regulating PI3K, STAT3 and Erk signaling [38]. These mechanisms may extend to HNSCC as well, since the involvement of IL-6 and PI3K signaling in HNSCC has been well documented by our laboratory and others [39], [40].

A major clinical challenge faced by oncologists is the lack of molecular markers to identify patients with oral leukoplakia lesions that are at high risk of transformation to malignancy. In this context, the significant increase in S100A7 expression observed in HNSCCs (67% cases) as compared to the oral leukoplakia lesions with dysplasia (44%) is an important finding of our study (p = 0.005, OR = 2.8, 95% CI = 1.4−5.7), suggesting that nuclear accumulation of S100A7 may be linked to increased risk of malignant transformation and might serve as a marker to identify the high-risk lesions. Nevertheless, this finding warrants confirmation in a longitudinal follow-up study of patients with oral leukoplakia lesions, to establish a possible link between nuclear S100A7 expression and risk of cancer development.

In our study, S100A7 was shown to be a prognostic factor for reduced survival of HNSCC patients. However, in contrast to these findings, the expression of this protein was associated with high differentiation. None of the poorly differentiated specimens (n = 6), expressed S100A7. This finding seems to be contradictory at a first glance. However, it is worthwhile to note that among the 100 HNSCC cases analyzed in our study, only 6 poorly differentiated tumors were analyzed; in comparison 45 well differentiated tumors were investigated. Thus larger number of poorly differentiated tumors needs to be analyzed to determine the correlation with S100A7 expression in a future study. Our study was also supported by an earlier report that showed association of nuclear S100A7 expression with well differentiated squamous cell carcinoma as compared to moderate and poorly differentiated squamous cell carcinomas [30]. In a parallel study using OSCC tissues sections, Kesting et al., [29] showed a significant correlation between increased S100A7 expression and tumor stage (I and II), well differentiated carcinomas and non-metastatic tumors, thus supporting our findings. Similar observations have been reported in bladder, breast and skin cancer [18], [29], [41]–[44]. S100A7 overexpression showed association with well differentiated squamous cell carcinoma of the bladder in comparison the less differentiated tumors. Similarly, S100A7 overexpression has been shown to be expressed in the superficial, differentiated region of the epithelium and its expression correlates with the degree of keratinocyte differentiation [18]. S100A7 expression is relatively low in normal, benign and atypical hyperplastic proliferative ductal lesions, high in the pre-invasive ductal carcinoma in situ (DCIS), but reduced in invasive carcinomas [41]–[44]. In skin tumors, it is absent in undifferentiated basalioma and strongly expressed in carcinoma in situ, as well as in keratoacanthoma and differentiated squamous cell carcinoma. Taken together, these findings clearly suggest the role of S100A7 in epithelial differentiation. In an attempt to explain the role of S100A7, Zhou et al. [30] demonstrated overexpression of S100A7 protein resulted in degradation of β-catenin by non-canonical pathway independent of GSK3β in oral cancer cells [30]. Further, overexpression of S100A7 inhibited cell proliferation in vitro but promoted tumor differentiation in an orthotopic xenograft oral cancer model, supporting our clinical findings. Thus, S100A7 overexpression suppresses tumor growth and invasion by negative regulation of β-catenin signaling. However, the exact mechanism explaining this biphasic role of S100A7 expression in proliferation in early stages but inducing differentiation in advanced stages in head and neck carcinogenesis remains to be completely understood and warrants further investigation.

Interestingly, S100A7 over expression (cytoplasmic/nuclear) has been associated with poor patient outcome in ER-negative invasive breast cancer patients [41], [45]. Notably, our study showed significance of nuclear S100A7 expression as a poor prognosticator of HNSCC (independent of other clinical and pathological parameters as revealed by Cox regression model). Further, our time dependent predictive analysis also revealed poor prognosis of HNSCC patients showing increased nuclear S100A7 expression. Taken together, these findings demonstrated the potential of nuclear S100A7 as a predictive marker for poor prognosis of HNSCC. Altered expression of several S100 proteins has also been associated with HNSCC [10], [25]–[28]. Recently, both S100A4 and S100A2 have been proposed as biomarkers of diagnostic and/or prognostic relevance [26], [46]. S100 family of proteins including S100A7 have been shown to form both homodimers and heterodimers interacting with c*jun* activation domain binding protein 1 (Jab1), Ran binding protein M (RanBPM), epidermal fatty acid binding protein (EFABP), and transglutaminase. Interactions with RanBPM have recently been shown to promote migration of renal cancer cells [47], suggesting the potential for S100A7 to influence invasive potential of cancer cells. Using 2D-gel electrophoresis, S100A7 was identified as a putative marker for lung SCC metastasis to brain [48]. Using in vivo models for HNSCC loss of both S100A7 and E-FABP have been shown to result in reduction of cell adhesion and increased cell motility, leading to distant metastasis [49]. Thus, we speculate that with a growing understanding of the role of S100A7 in cell migration, invasion and proliferation pathways, S100A7 may serve as a therapeutic target for the treatment of inflammation and cancer.

In conclusion, S100A7 was shown to be expressed in oral lesions in early stages, prior to onset of dysplasia and in frank tumors. Its subcellular localization, suggests that nuclear S100A7 may be associated with increased risk of transformation of oral pre-malignant lesions and recurrence in HNSCC. Furthermore, increased nuclear accumulation of S100A7 in HNSCC as compared to dysplastic lesions warrants a large-scale longitudinal study of patients with dysplasia to evaluate its potential as a determinant of increased risk of transformation of oral premalignant lesions and recurrence in HNSCC.

## Materials and Methods

### Patients and clinicopathological data collection, tissue specimens

The Institutional Human Ethics Committee of the All India Institute of Medical Sciences (AIIMS), New Delhi, India, approved this study prior to its commencement. Tissue specimens were obtained by diagnostic or therapeutic procedures from 166 patients with oral lesions- clinically defined leukoplakia [with squamous cell hyperplasia (n = 116) or with dysplasia (n = 50)] attending the Outpatient Clinic of the Departments of Surgical Disciplines and Otorhinolaryngology, AIIMS, and from 100 HNSCC patients undergoing curative cancer surgery during the period 2002–2007, after obtaining the patients' written consent. Wherever possible, non-malignant tissues (n = 43) were taken each from a site distant from the surgically resected HNSCC. Non-malignant oral tissues (n = 57) were also collected from the patients attending the Outpatient Department of Dental Surgery for tooth extraction, after obtaining the patients' written consent. Taken together, these 100 non-malignant oral tissues with histological evidence of normal epithelia constituted the normal group. After excision, tissues were immediately snap-frozen in liquid nitrogen and stored at −80°C in the Research Tissue Bank till further use; one part of the tissue was collected in 10% formalin and embedded in paraffin for histopathological and immunohistochemical analyses. Histologically confirmed oral normal epithelia, squamous cell hyperplasia, dysplasia, and HNSCC as revealed by H & E staining were used for immunohistochemistry [7]. Patient demographic, clinical, and pathological data were recorded in a pre-designed performa as described previously [7], [50]. The information documented included clinical TNM staging (tumor, node, and metastasis based on the Union International Center le Cancer TNM classification of malignant tumors 2002), site of the lesion, histopathological differentiation, age, gender, and tobacco consumption habits.

### Follow-up Study

Seventy-seven HNSCC patients who underwent treatment from 2002–2007 were investigated and evaluated in the head-and-neck cancer follow-up clinic at regular time intervals. Survival status of the HNSCC patients was verified and updated from the records of the Tumor Registry, Institute Rotary Cancer Hospital, AIIMS, as of December 2009. HNSCC patients were monitored for a maximum period of 83 months. Disease-free survivors were defined as patients free from clinical and radiological evidence of local, regional, or distant relapse at the time of the last follow-up [50], [51]. Loco-regional relapse/death were observed in 51 of 77 (66%) patients monitored during the follow-up. Twenty six patients who did not show recurrence were alive until the end of the follow-up period. Only disease-free survival was evaluated in the present study, as the number of deaths due to disease progression did not allow a reliable statistical analysis. Disease-free survival was expressed as the number of months from the date of surgery to loco-regional relapse/death.

### Immunohistochemistry

Paraffin-embedded sections (5 µm) of human oral non-malignant tissues (n = 100), oral lesions [squamous cell hyperplasia (n = 116) or with dysplasia (n = 50)] and HNSCC (n = 100) were collected on gelatin-coated slides. In brief, the sections were deparaffinized in xylene, hydrated in gradient alcohol, and pre-treated in a microwave oven for 10 min at 800 W and 5 min at 480 W in citrate buffer (0.01 M, pH = 6.0) for antigen retrieval. The sections were incubated with hydrogen peroxide (0.3% v/v) in methanol for 30 min to quench the endogenous peroxidase activity, followed by blocking with 1% bovine serum albumin (BSA) to preclude nonspecific binding. Thereafter, the slides were incubated with mouse monoclonal anti-S100A7 antibody (0.5 µg/ml, sc-52948, Santa Cruz Biotechnology, CA) for 16 h at 4°C. The primary antibody was detected using the streptavidin-biotin complex with the Dako LSAB plus kit (Dako Cytomation, Glostrup, Denmark) and diaminobenzidine as the chromogen as described before [7], [8]. In the negative control tissue sections, the primary antibody was replaced by isotype specific non-immune mouse IgG. A section from estrogen receptor (ER)-negative breast cancer tissue was used as a positive control in each batch of immunohistochemistry.

### Evaluation of immunohistochemical staining

Each slide was evaluated for S100A7 immunostaining using a semi-quantitative scoring system for both staining intensity and the percentage of positive epithelial cells [8]. For S100A7 protein expression, sections were scored as positive if epithelial cells showed immunopositivity in the nucleus/cytoplasm when observed independently by four of us (SCT, AM, JK, SDG), who were blinded to the clinical outcome (the slides were coded and the scorers did not have prior knowledge of the local tumor burden, lymphonodular spread, and grading of the tissue samples). The tissue sections were scored based on the % of immunostained cells as: 0–10% = 0; 10–30% = 1; 30–50% = 2; 50–70% = 3 and 70–100% = 4. Sections were also scored semi-quantitatively on the basis of staining intensity as negative  = 0; mild  = 1; moderate  = 2; intense  = 3 [50], [51]. Finally, a total score was obtained by adding the score of percentage positivity and intensity. In cases where both nuclear and cytoplasmic immunoreactivity was observed, the nuclear and cytoplasmic staining was scored independently. The scoring by the four observers was discrepant in about 5% cases and a consensus on the final result was reached by re-evaluation of these slides and discussion.

### Statistical Analyses

The immunohistochemical data were subjected to statistical analyses using the SPSS 10.0 software (Chicago). Sensitivity and specificity were calculated and quantified using receiver operating characteristic (ROC) analyses. The predictive value (PV) describes the proportion of correctly classified cases. Based on sensitivity and specificity values for S100A7, a cutoff ≥3 was defined as positive criterion for both cytoplasmic and nuclear S100A7 immunopositivity for statistical analyses. The relationships between S100A7 protein expression and clinicopathological parameters were tested using Chi-Square and Fischer's exact test. Two-sided p values were calculated and p<0.05 was considered to be significant. Similarly, positive predictive value (PPV) was calculated for oral leukoplakia and HNSCC with respect to normal tissues.

The correlation of S100A7 staining with patient survival was evaluated using life tables constructed from survival data with Kaplan-Meier plots [50], [51]. Multivariate analysis was carried out using Cox regression model. The systematic and rigorous assessment of Positive and Negative Predictive Values (PPV and NPV respectively) for prognostic biomarkers was carried out as described earlier by us [50]. For the follow-up study of HNSCC, let T denote the failure time, i.e., the first time recurrence is diagnosed after surgical removal of the tumor. For these data, the positive and negative predictive values as functions of time are defined as follows:

PPV_tumor_(t)  =  Prob (T ≤ t AND Recurrence| S100A7 (nuclear) ≥3);

NPV_tumor_(t)  =  Prob (T > t OR No Recurrence| S100A7 (nuclear) <3); 0≤ t ≤83

These probabilities are estimated from the observed accumulated incidences over the respective time periods.

### Verification of S100A7 expression using Reverse Transcription-PCR and Western blotting

Representative frozen tissue specimens of histologically confirmed oral normal tissues (n = 5), squamous cell hyperplasia (n = 5), dysplasia (n = 5), and HNSCC (n = 5) were used for extraction of total RNA using the TRI reagent (Sigma, MO) as previously described [7], [52]. First-strand cDNA was synthesized using 2 µg RNA with oligo dT as the primer with MMLV reverse transcriptase. PCR was carried out using S100A7 specific primers forward (5′-CTTCCTTAGTGCCTGTGACAAAAA-3′) and reverse (5′-AAGGACAGAAACTCAGAAAA ATCAATCT-3′) and PCR product was visualized on agarose gel with UV light. Western blotting was carried out using whole-cell lysates in same tissue samples as used for RT-PCR as described earlier [7], [8].
